# Relationship between peri-coronary inflammation and coronary vascular function in patients with suspected coronary artery disease

**DOI:** 10.3389/fcvm.2024.1303529

**Published:** 2024-02-08

**Authors:** Mengyu Chen, Bing Liu, Xu Li, Dong Li, Lijuan Fan

**Affiliations:** ^1^ Department of Radiology, TEDA International Cardiovascular Hospital, Cardiovascular Clinical College of Tianjin Medical University, Tianjin, China; ^2^ Department of Radiology, TEDA International Cardiovascular Hospital, Tianjin University, Tianjin, China; ^3^ Tianjin Key Laboratory of Molecular Regulation of Cardiovascular Diseases and Translational Medicine, Tianjin, China; ^4^ Department of Radiology, Tianjin Medical University General Hospital, Tianjin, China

**Keywords:** coronary flow reserve, peri-coronary adipose tissue, fat attenuation index, coronary artery disease, perivascular inflammation

## Abstract

**Background:**

In this study, we aim to investigate the relationship between the attenuation of peri-coronary adipose tissue (PCAT) in patients with suspected coronary artery disease (CAD) and the assessment of coronary vascular functions using coronary flow reserve (CFR).

**Methods:**

We included 364 patients who underwent ^13^N-NH_3_ positron emission tomography/computed tomography and coronary computed tomography angiography (CCTA). We determined the relationship between fat attenuation index (FAI), PCAT volume, and other qualitative CT-derived anatomic parameters with CFR.

**Results:**

We detected a decrease in CFR (<2.5) in 206 (57%) patients. At the patient level, those with reduced CFR showed a significantly higher prevalence of diffused atherosclerosis (41% vs. 23%; *P* < 0.001) and higher FAI (−75.5 HU vs. −77.1 HU; *P* = 0.014). In patients without obstructive CAD, FAI was significantly higher in those with reduced CFR (−75.5 HU vs. −77.7 HU, *P* = 0.026). On the vessel level, 1,092 vessels were analyzed, and 642 (59%) exhibited reduced CFR. The vessels with reduced CFR presented a significantly higher prevalence of obstructive CAD (37% vs. 26%; *P* < 0.001), diffused atherosclerosis (22% vs. 11%; *P* < 0.001), low-attenuation plaque (6% vs. 3%; *P* = 0.030), and positive remodeling (7% vs. 2%; *P* = 0.001). FAI was higher in vessels with reduced CFR (−80.8 HU vs. −81.8 HU; *P* = 0.045) than in normal CFR. In the patient-level analysis, obstructive CAD, diffused atherosclerosis, and FAI were independently linked with CFR. FAI was still associated with global CFR after adjusting for traditional risk factors (age, hypertension, diabetes, hyperlipidemia, and smoking). FAI remained independently associated with reduced CFR in patients without obstructive CAD.

**Conclusions:**

Coronary perivascular inflammation evaluated by CCTA was independently associated with coronary vascular function. In patients without obstructive CAD, FAI was higher in the presence of reduced CFR. Altogether, FAI can help reveal microcirculatory damage in patients who do not exhibit epicardial artery stenosis.

## Introduction

Peri-coronary adipose tissue (PCAT) attenuation, derived from coronary computed tomography angiography (CCTA), is linked with coronary artery inflammation (1). The changes in the PCAT composition from lipid to aqueous can reveal inflammation in the surrounding coronary arteries.

Fat attenuation index (FAI) is a novel imaging biomarker for the presence of coronary artery inflammation (2). A study using two large cohorts reported the associations between increased cardiac mortality and higher FAI, confirming the prognostic value of FAI (3). In addition, FAI is also a novel parameter indicating changes in coronary artery hemodynamics. A previous study showed that perivascular FAI is significantly higher for flow-limiting lesions than non-flow-limiting lesions (4). Nevertheless, the specificity of perivascular FAI in identifying flow-limiting lesions is relatively low. Furthermore, the combination of perivascular FAI and other CCTA characteristics, such as diameter stenosis, can improve the accuracy of diagnosing ischemic coronary artery stenosis.

Positron emission tomography (PET) is the most accurate non-invasive method for evaluating myocardial blood flow (MBF), which can obtain MBF in both cardiac load and resting states. Coronary flow reserve (CFR) is the ratio of MBF during maximum coronary congestion to resting, which is considered a powerful indicator of the myocardial reserve capacity (5). The presence of abnormal CFR indicates the presence of coronary artery stenosis and coronary artery dysfunction (5, 6). CFR can reveal epicardial coronary artery stenosis and impaired vasodilatory capacity through diffused atherosclerotic (DA) changes (7). Nevertheless, it is challenging to determine whether reduced CFR is caused by epicardial coronary stenosis or microvascular dysfunction. A previous study showed that abnormal CFR (<2.5) indicates microvascular dysfunction in the absence of significant stenosis of the epicardial coronary artery (<50%) in patients (8). Presently, most studies focus on the effect and prediction of peri-coronary inflammation on the hemodynamics of epicardial coronary arteries. However, the effect of microvascular function is not well reported (9). In this study, we investigated the association between FAI and coronary vascular functions, particularly microvascular function.

## Methods

### Study population

**Figure 1:**
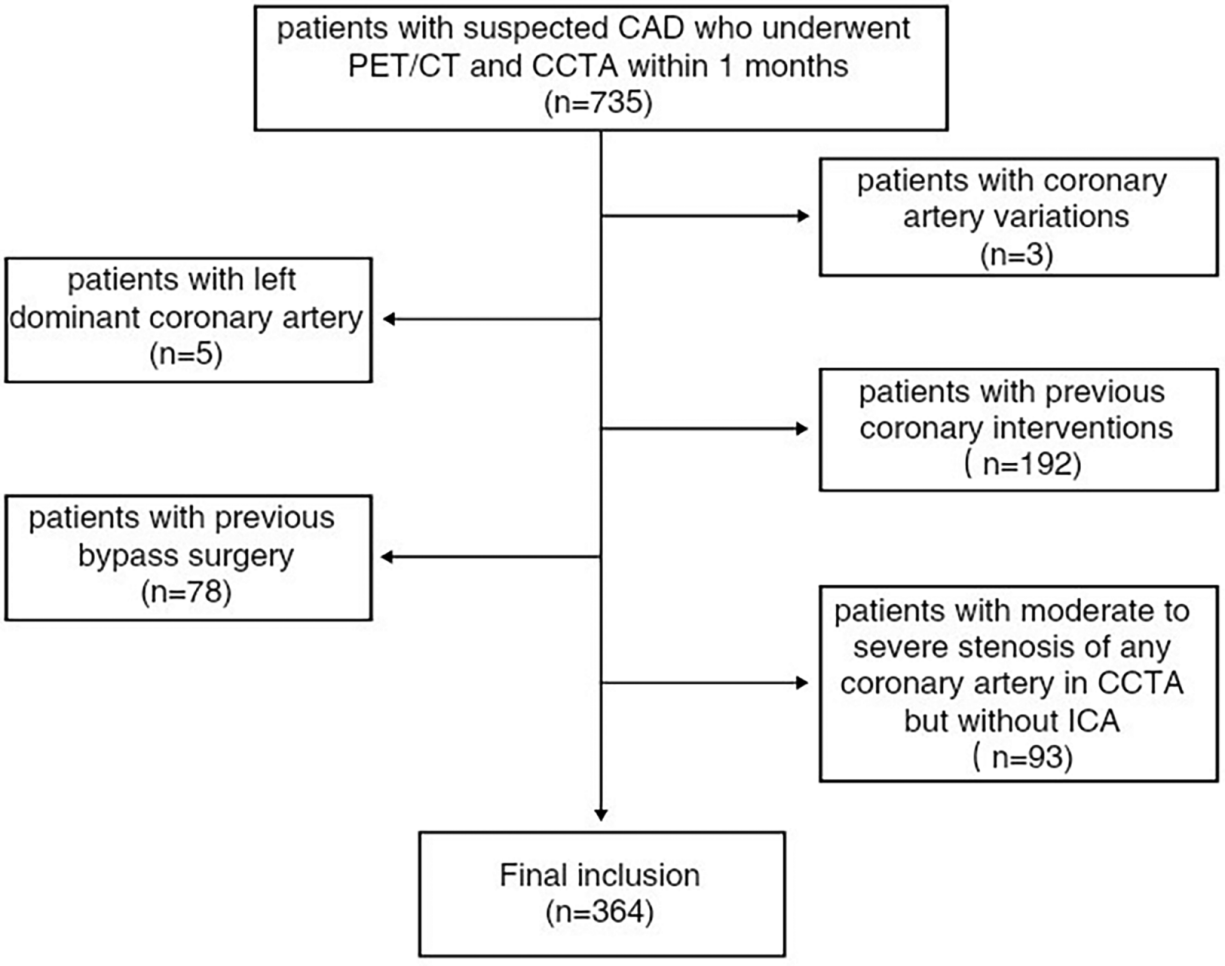
Inclusion and exclusion criteria for the study population. CAD, coronary artery disease; CCTA, coronary computed tomography angiography; ICA, invasive coronary angiography; PET/CT, positron emission tomography/computed tomography.

We included consecutive patients with suspected coronary artery disease (CAD) who visited our institute from January 2021 to June 2023 and underwent ^13^N-NH_3_ positron emission tomography/computed tomography (PET/CT) and CCTA within 1 month. Patients with moderate to severe stenosis of any coronary artery revealed by CCTA required further invasive coronary angiography (ICA). The exclusion criteria were as follows: (I) patients with coronary artery variations, such as origin variation, conformational variation, and termination abnormality; or (II) patients with left dominant coronary artery; or (III) patients with concomitant cardiomyopathy; or (IV) patients with any previous coronary interventions or bypass surgery; or (V) patients with incomplete baseline clinical data; or (VI) patients with moderate to severe stenosis of any coronary artery in CCTA but without ICA; or (VII) patients with a significantly impaired image quality on CCTA. Lastly, 364 patients were enrolled in this study (Figure 1). The need for written informed consent was waived due to the retrospective design of the study. The hospital ethics committee approved this study.

### CCTA examination

CCTA scans were performed using a GE 256-slice computed tomography scanner (Revolution, GE, USA). The CTA scan adopted a prospective electrocardiogram gating protocol, and the scan parameters were set as follows: tube voltage of 100 or 120 kV and tube current of 300–700 mA, which was based on the body mass index. Patients with elevated heart rates should have a heart rate below 75 beats/min prior to administering a contrast injection. A bolus of iodine contrast medium (Ioversol solution), 60–70 ml, was injected at a rate of 4.5–5 ml/s into the antecubital vein.

### PCAT attenuation analysis

PCAT was defined as the radial distance from the vessel wall equal to the vessel diameter for all voxels in the range of −190 to −30 HU (10). PCAT attenuation was evaluated in the proximal 40-mm segment of the left anterior descending artery (LAD) and left circumflex artery (LCX) and the proximal 10- to 50-mm segment of the right coronary artery (RCA) (10). PCAT attenuation was calculated based on the average attenuation of the perivascular adipose tissue. Semiautomated software (CoronaryDoc, Shukun Technology, China) was used to perform PCAT attenuation analysis as previously described (11). The global FAI was defined as the lowest value among the three major coronary arteries, whereas the global PCAT volume was calculated as the average of the three arteries. FAI was considered high at >−70.1 HU.

### Image analysis of CCTA

Two radiologists (with 10 years and 3 years of diagnostic experience in cardiac imaging) independently analyzed all CCTA images. In cases of differences of opinion, consent was achieved after a joint discussion. The diagnosis of mild stenosis lesions was accurate due to the high negative predictive value of CCTA. A subsequent ICA examination was performed following the detection of ≥50% stenosis in the three major coronary arteries on CCTA. Furthermore, the final degree of stenosis was based on ICA. Curved planar reformation (CPR) and axial views were used to analyze the CCTA images. The stenosis severity was categorized into the following five groups: 0%, 1%–49%, 50%–69%, 70%–90%, and >90%. Obstructive CAD was considered when luminal stenosis was ≥50% (12). Simultaneously, we considered the effect of the presence of diffused atherosclerosis (DA). DA was defined as a single or tandem lesion measuring ≥2 cm or multifocal plaques diffusely distributed along the vascular wall with or without calcification (7). The characteristics of high-risk plaque were as follows: low-attenuation plaque (any voxel <30 HU), positive remodeling (ratio of cross-sectional lesion area by the area of a proximal reference point >1.1), spotty calcification (small calcified portion <3 mm length and presenting <90° of vessel circumference), and napkin ring sign (plaque core with low CT attenuation surrounded by a ring-like peripheral of higher CT attenuation) (13). Patients with at least two characteristics of high-risk plaque were considered to have high-risk plaques (14).

### PET/CT image acquisition and analysis

The patients underwent fasting for at least 4 h and refrained from caffeine for at least 24 h prior to the procedure. All patients received an adequate history inquiry and informed consent. An intravenous line was placed in the radial vein of the patients, and a low-dose CT scan (120 kV, 20 mA) was performed for attenuation correction. Subsequently, 10 mCi (370–555 MBq) of ^13^N-ammonia was injected intravenously as a bolus 10 s prior to the start of a 10 min dynamic PET acquisition in 3D list-mode format. After ^13^N-ammonia dynamic PET acquisition and a 2 min wait, ECG-gated perfusion ^13^N-ammonia PET with 8 frames per cardiac cycle was performed for 8 min. The interval between resting and stress imaging was at least 40 min, and 0.16 mg/kg/min of adenosine was intravenously infused for 6 min as a vasodilator. Then, ^13^N-ammonia was injected 3 min after the initiation of the infusion. Stress image acquisition was performed with the same dose and protocol as that for resting images. All PET scanning was performed using a PET/CT scanner (GE Discovery NM690).

QPET (Cedars-Sinai, LA, USA) was used for processing PET/CT images. We acquired the rest and stress MBF of three coronary artery perfusion regions and calculated the zoning and left ventricular coronary flow reserve (LV-CFR) (as the ratio of the stress/rest MBF). CFR was considered reduced when it was less than 2.5 (8, 15).

### Baseline clinical characteristics

Baseline characteristics were retrospectively acquired from the electronic medical record system, which included the demographic characteristics (gender, age, and body mass index) and conventional high-risk factors of CAD (hyperlipidemia, hypertension, diabetes, and current smoking) of patients.

### Statistical analysis

SPSS Statistics 23.0 software (IBM Corporation, Armonk, NY, USA) was used to perform statistical analyses. The continuous variables with a normal distribution are presented as mean ± standard deviation (SD), whereas the median (25th and 75th) is used to express non-normal distributions. The independent *t*-test or Mann–Whitney *U* test was performed to compare the quantitative parameters of the two groups. Categorical variables are expressed as absolute frequencies and percentages and compared using the *χ*^2^ test. In the patient-level analysis, logistic univariate and multivariate regression analyses were performed to determine the associations between global CFR and PCAT volume, FAI, diffused atherosclerosis, vulnerable plaques, and CAD risk factors. Pearson's correlation analysis was performed to evaluate the correlation between CFR, rest MBF, and stress MBF with FAI. The receiver operating characteristic (ROC) curve was performed to determine the optimal FAI cut-off value for defining reduced CFR in total, obstructive, and non-obstructive lesions. A *P*-value of <0.05 was considered statistically significant.

## Results

### Study population

A total of 364 participants with 1,092 vessels were retrospectively reviewed in the present study, and their clinical and imaging characteristics are displayed in Table 1. The mean age of the included patients was 60 ± 11 years, and 179 patients (49%) were females. A total of 205 (56%) patients suffered from hypertension, and 69 (19%) patients had diabetes. A total of 205 (56%) patients presented obstructive CAD, and 122 (34%) presented DA. The global FAI was −76.2 ± 6.4 HU, and the PCAT volume was 1,866 ± 373 mm^3^.

**Table 1:** Patient characteristics.

*N* = 364	
Demographics	
Age, years	60 ± 11
Female, *n* (%)	179 (49%)
Body mass index, kg/m^2^	25.6 ± 3.9
CAD risk factors, *n* (%)	
Hypertension	205 (56%)
Hyperlipidemia	115 (32%)
Diabetes	69 (19%)
Smoking	122 (34%)
CCTA/ICA, *n* (%)	
0%	88 (24%)
1%–49%	71 (20%)
50%–69%	39 (11%)
70%–90%	93 (25%)
>90%	73 (20%)
Diffused atherosclerosis	122 (34%)
FAI, HU	−76.2 ± 6.4
PCAT volume, mm^3^	1,866 ± 373
PET/CT	
Rest MBF, ml/min/g	0.87 (0.79, 0.92)
Stress MBF, ml/min/g	1.93 (1.37, 2.55)
CFR	2.26 (1.70, 3.04)

CAD, coronary artery disease; CCTA, coronary computed tomography angiography; ICA, invasive coronary angiography; FAI, fat attenuation index; PCAT, peri-coronary adipose tissue; PET/CT, positron emission tomography/computed tomography; MBF, myocardial blood flow; CFR, coronary flow reserve.

### Relationship between perivascular inflammation and global CFR at the patient level

**Figure 2:**
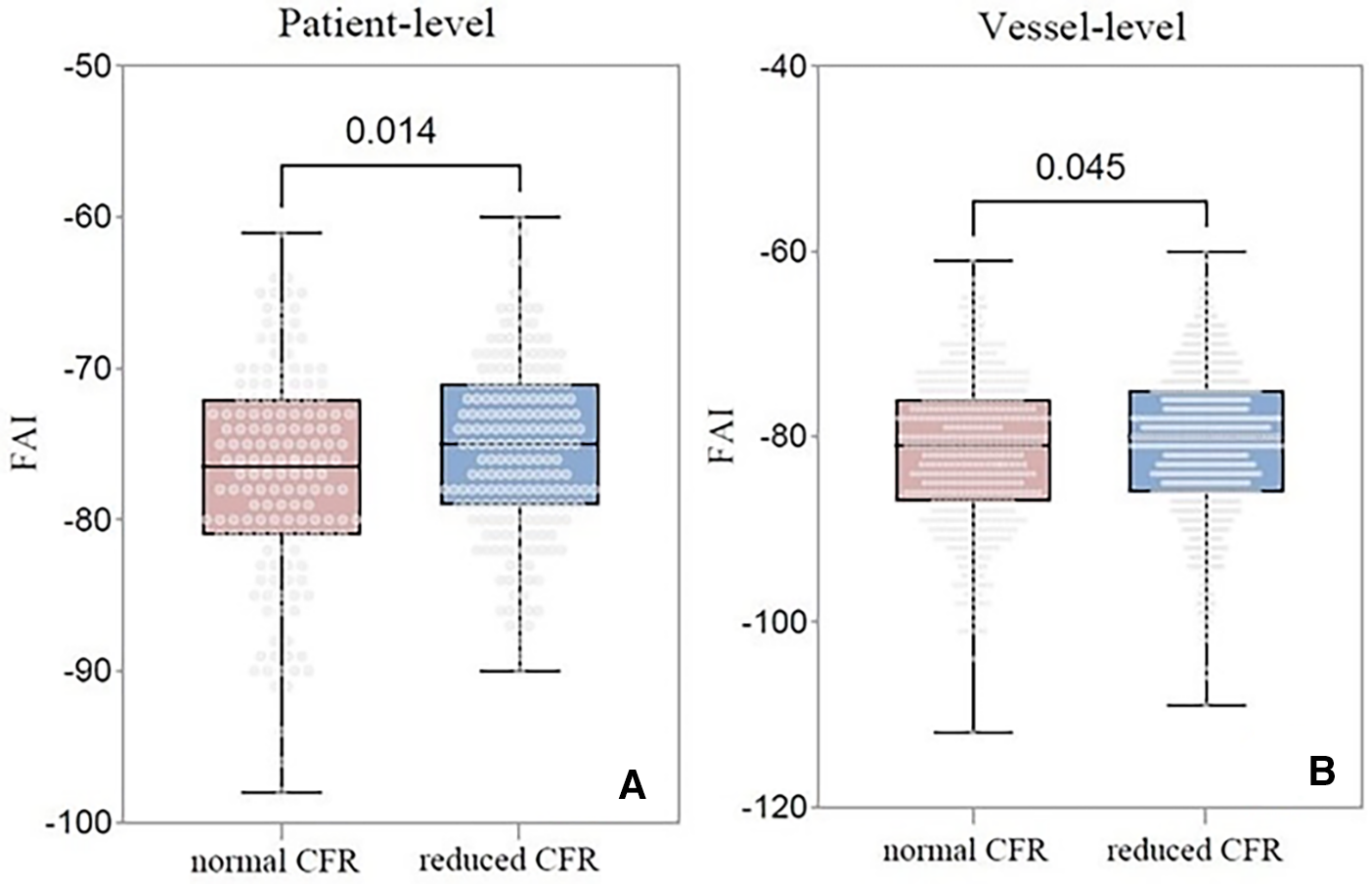
Comparison of the FAI between normal CFR group and reduced CFR group. (**A**) Comparison at the patient level; (**B**) Comparison at the vessel level. FAI, fat attenuation index; CFR, coronary flow reserve; CAD, coronary artery disease.

**Figure 3:**
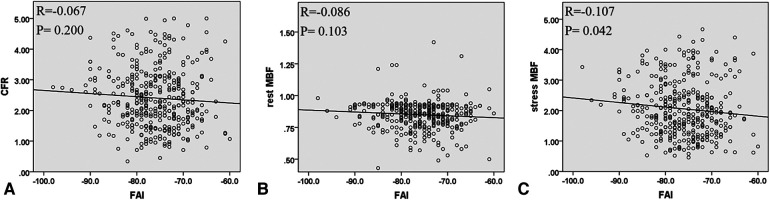
The correlation between FAI with CFR, rest MBF, and stress MBF at the patient level. (**A**) FAI and CFR, (**B**) FAI and rest MBF, and (**C**) FAI and stress MBF. FAI, fat attenuation index; CFR, coronary flow reserve; MBF, myocardial blood flow.

At the patient level, a decrease in CFR was observed in 206 (56%) patients. The clinical and imaging characteristics of patients with a normal or reduced CFR are listed in Table 2. Compared with patients with normal CFR, those with reduced CFR showed a significantly higher prevalence of obstructive CAD (65% vs. 44%; *P* < 0.001) and DA (41% vs. 23%; *P* < 0.001). Moreover, the FAI was higher in patients with reduced CFR (−75.5 HU vs. −77.1 HU; *P* = 0.014) than in those with normal CFR (Figure 2A). No significant difference was observed in the rest MBF between the two groups, whereas the stress MBF of the reduced CFR group was significantly lower (1.45 vs. 2.68; *P* < 0.001) than that of the normal CFR group. A weak negative correlation was observed between the FAI and the stress MBF, whereas no significant correlation was observed between the rest MBF and the FAI (Figure 3).

**Table 2:** Clinical and imaging findings in the study population.

	CFR ≥ 2.5	CFR<2.5	*P*-value	*χ* ^2^
	(*n* = 158)	(*n* = 206)		
Age, years	60 ± 10	60 ± 11	0.484	
Female, *n* (%)	87 (55%)	92 (45%)	0.049*	3.872
Body mass index, kg/m^2^	25.2 ± 4.2	25.8 ± 3.7	0.189	
Hypertension, *n* (%)	87 (55%)	118 (57%)	0.672	0.179
Hyperlipidemia, *n* (%)	51 (32%)	64 (31%)	0.806	0.061
Diabetes, *n* (%)	26 (16%)	43 (21%)	0.286	1.136
Smoking, *n* (%)	46 (29%)	76 (37%)	0.119	2.428
Obstructive CAD, *n* (%)	70 (44%)	134 (65%)	0.000*	15.620
Diffused atherosclerosis, *n* (%)	37 (23%)	85 (41%)	0.000*	12.778
High-risk plaques, *n* (%)	18 (11%)	29 (14%)	0.449	0.537
FAI, HU	−77.1 ± 7.0	−75.5 ± 5.8	0.014*	
PCAT volume, mm^3^	1,859 ± 380	1,872 ± 368	0.756	
Rest MBF, ml/min/g	0.87 (0.81, 0.92)	0.86 (0.78, 0.92)	0.201	
Stress MBF, ml/min/g	2.68 (2.36, 3.36)	1.45 (1.14, 1.77)	0.000*	

CAD, coronary artery disease; FAI, fat attenuation index; PCAT, peri-coronary adipose tissue; MBF, myocardial blood flow; CFR, coronary flow reserve.

* *P* < 0.05.

**Figure 4:**
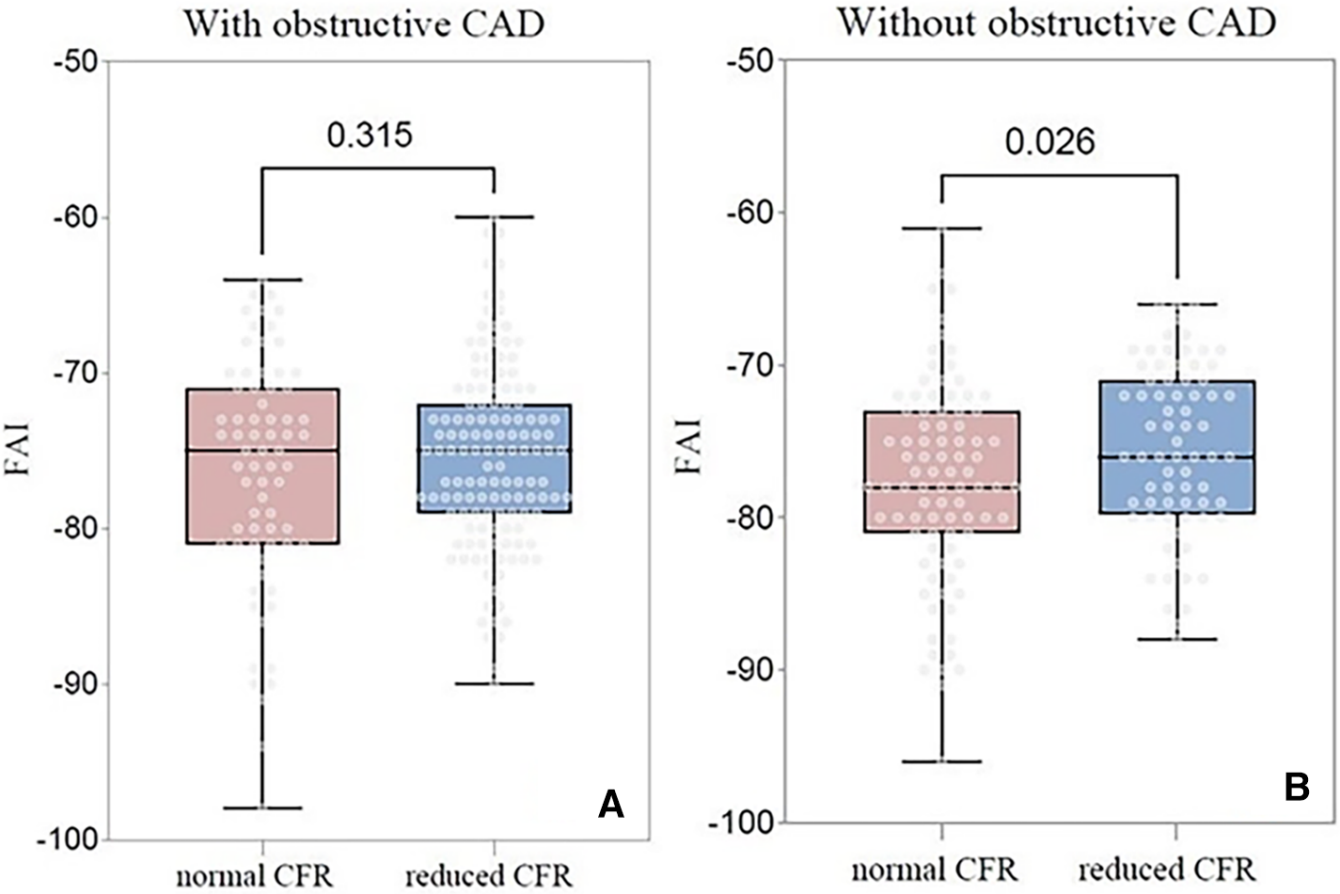
Comparison of the FAI between the normal CFR group and the reduced CFR group at the patient level. (**A**) Comparison in patients with obstructive CAD; (**B**) Comparison in patients without obstructive CAD. FAI, fat attenuation index; CFR, coronary flow reserve; CAD, coronary artery disease.

In patients with obstructive CAD, no significant difference was observed between the FAI of the patients with a normal and reduced CFR (−76.4 HU vs. −75.4 HU; *P* = 0.315) (Figure 4A). Conversely, in patients without obstructive CAD, FAI was significantly higher in patients with reduced CFR (−75.5 HU vs. −77.7 HU; *P* = 0.026) than in those with normal CFR (Figure 4B). However, no significant difference was observed between the global CFR of patients with high and low FAI among patients with or without obstructive CAD.

The results of the logistic regression analysis considering reduced CFR (<2.5) as a dependent variable in all patients are presented in Table 3. No significant correlation was observed among demographics (age, gender, and body mass index), CAD risk factors (hypertension, hyperlipidemia, diabetes, and smoking), high-risk plaques, PCAT volume, and reduced CFR. The univariate analysis results showed that obstructive CAD, DA, and FAI were related to reduced CFR. However, the multivariable analysis results showed that only obstructive CAD (*P* = 0.027) and FAI (*P* = 0.035) were independently related to reduced CFR.

**Table 3:** Logistic regression analyses with CFR < 2.5 as a dependent variable in the total patients.

	Univariable analysis		Multivariable analysis	
	Odds ratio	*P*-value	Odds ratio	*P*-value
Age, years	1.007	0.483		
Female, *n* (%)	0.659	0.050		
Body mass index, kg/m^2^	1.038	0.182		
Hypertension, *n* (%)	1.094	0.672		
Hyperlipidemia, *n* (%)	0.946	0.806		
Diabetes, *n* (%)	1.338	0.287		
Smoking, *n* (%)	1.423	0.120		
Obstructive CAD, *n* (%)	2.340	0.000*	1.831	0.027*
Diffused atherosclerosis, *n* (%)	2.297	0.000*	1.483	0.186
High-risk plaques, *n* (%)	1.274	0.450		
FAI, HU	1.047	0.015*	1.037	0.035*
PCAT volume, mm^3^	1.000	0.755		

CFR, coronary flow reserve; CAD, coronary artery disease; FAI, fat attenuation index; PCAT, peri-coronary adipose tissue.

* *P* < 0.05.

The results of the logistic regression analysis considering reduced CFR (<2.5) as a dependent variable in patients without obstructive CAD are presented in Table 4. The univariate analysis results showed that only FAI was related to reduced CFR in patients without obstructive CAD (*P* = 0.029). Based on the univariate analysis results and existing knowledge, female sex, hyperlipidemia, smoking, and FAI were selected as independent variables for the multivariate analysis, which showed that FAI was independently related to reduced CFR (*P* = 0.045).

**Table 4:** Logistic regression analyses with CFR < 2.5 as a dependent variable in patients without obstructive CAD.

	Univariable analysis		Multivariable analysis	
	Odds ratio	*P*-value	Odds ratio	*P*-value
Age, years	0.984	0.270		
Female, *n* (%)	1.608	0.156	1.012	0.977
Body mass index, kg/m^2^	1.020	0.605		
Hypertension, *n* (%)	1.208	0.555		
Hyperlipidemia, *n* (%)	1.633	0.178	1.826	0.126
Diabetes, *n* (%)	1.466	0.448		
Smoking, *n* (%)	2.053	0.105	1.678	0.291
FAI, HU	1.060	0.029*	1.059	0.045*
PCAT volume, mm^3^	0.999	0.175		

CFR, coronary flow reserve; CAD, coronary artery disease; FAI, fat attenuation index; PCAT, peri-coronary adipose tissue.

* *P* < 0.05.

### Relationship between perivascular inflammation and CFR at the vessel level

**Figure 5:**
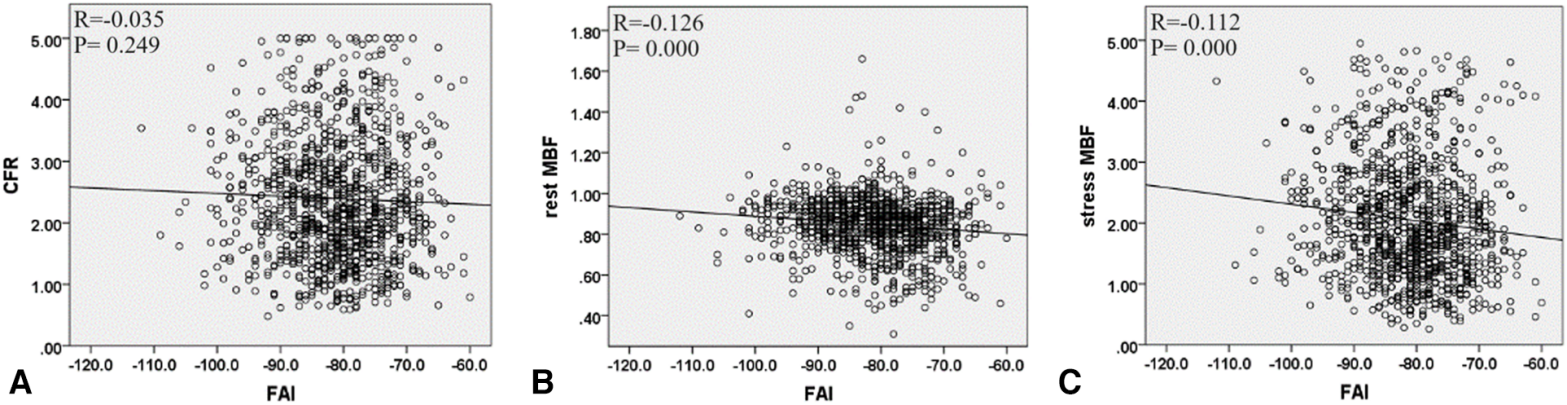
The correlation between FAI and CFR, rest MBF, and stress MBF at the vessel level. (**A**) FAI and CFR, (**B**) FAI and rest MBF, and (**C**) FAI and stress MBF. FAI, fat attenuation index; CFR, coronary flow reserve; MBF, myocardial blood flow.

A total of 1,092 vessels were analyzed, of which 642 (59%) exhibited reduced CFR. The imaging results of these vessels are presented in Table 5. Compared with vessels with normal CFR, vessels with reduced CFR presented a significantly higher prevalence of obstructive CAD (37% vs. 26%; *P* < 0.001), diffused atherosclerosis (22% vs. 11%; *P* < 0.001), low-attenuation plaque (6% vs. 3%; *P* = 0.030), and positive remodeling (7% vs. 2%; *P* = 0.001). Moreover, vessels with reduced CFR showed a higher FAI (−80.8 HU vs. −81.8 HU; *P* = 0.045) than that showed by vessels with normal CFR (Figure 2B). A weak negative correlation was observed between the FAI and the rest and stress MBF (Figure 5).

**Table 5:** Imaging findings in vessels with normal or reduced CFR.

	CFR ≥ 2.5	CFR < 2.5	*P*-value	*χ* ^2^
	(*n* = 450)	(*n* = 642)		
Obstructive CAD, *n* (%)	115 (26%)	239 (37%)	0.000*	16.451
Luminal stenosis				
0%	253 (56%)	258 (40%)		
1%–49%	83 (18%)	145 (23%)		
50%–69%	46 (10%)	78 (12%)		
70%–90%	48 (11%)	94 (15%)		
>90%	21 (5%)	67 (10%)		
FAI, HU	−81.8 ± 8.1	−80.8 ± 7.9	0.045*	
PCAT volume, mm^3^	1,872 ± 552	1,865 ± 573	0.829	
Diffused atherosclerosis, *n* (%)	50 (11%)	142 (22%)	0.000*	22.120
High-risk plaques, *n* (%)	21 (5%)	36 (6%)	0.491	0.473
Low-attenuation plaque	15 (3%)	41 (6%)	0.030*	4.715
Positive remodeling	11 (2%)	45 (7%)	0.001*	11.331
Spotty calcification	16 (4%)	32 (5%)	0.257	1.285
Napkin ring sign	9 (2%)	22 (3%)	0.162	1.953

CAD, coronary artery disease; FAI, fat attenuation index; PCAT, peri-coronary adipose tissue; CFR, coronary flow reserve.

* *P* < 0.05.

**Figure 6:**
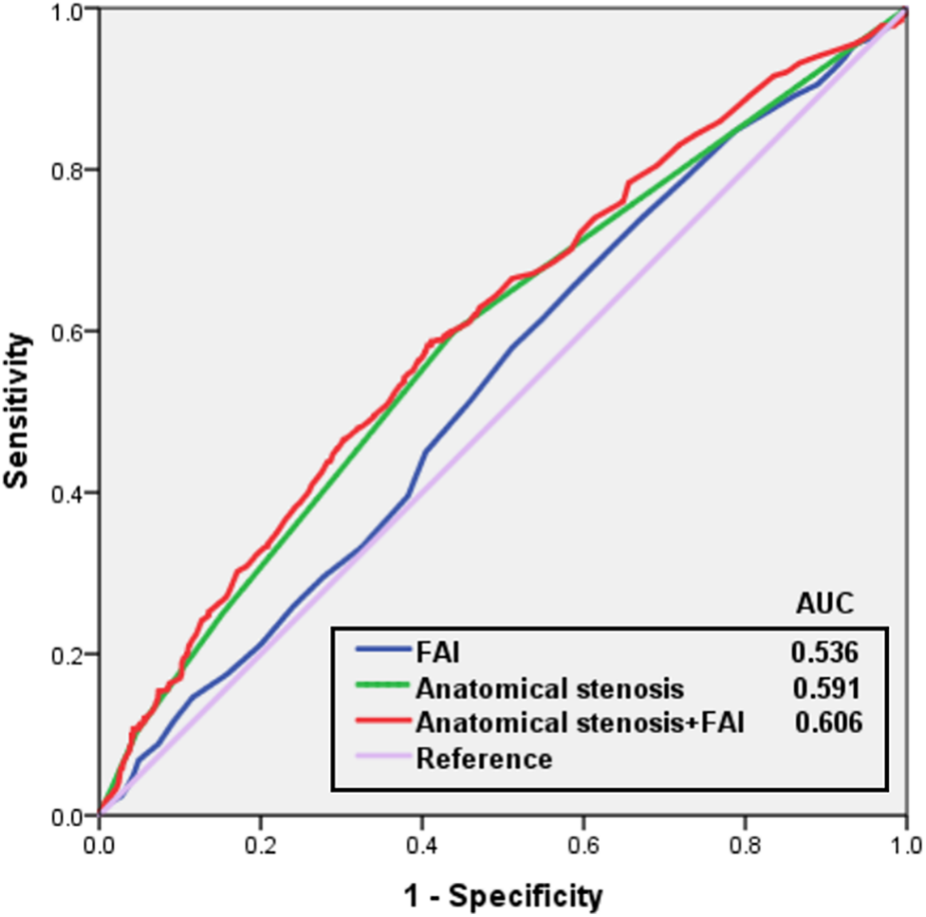
Receiver operating characteristic curves for anatomical stenosis and FAI in predicting reduced CFR. Cut-off value of −84.5 HU for FAI according to the Youden index. FAI, fat attenuation index; AUC, area under the curve.

An ROC curve was generated to determine the optimal FAI cut-off value in order to define CFR < 2.5. The Youden index showed that the optimal cut-off values of FAI for total, obstructive, and non-obstructive lesions were −84.5 HU, −87.5 HU, and −82.5 HU, respectively. Moreover, the ROC curve was used to analyze the diagnostic efficacy of anatomical stenosis, FAI, and their combination in identifying CFR < 2.5, and the results showed that the areas under the curve (AUCs) for anatomical stenosis, FAI, and their combination were 0.591 [95% confidence interval (CI), 0.561–0.621], 0.536 (95% CI, 0.506–0.566), and 0.606 (95% CI, 0.577–0.635), respectively (Figure 6). Although the AUC of the combination was higher than that of anatomical stenosis, no significant differences were observed between them (*P* = 0.067).

## Discussion

The present study showed that the FAI was higher in the reduced CFR group than in the normal CFR group at both patient and vessel levels. Furthermore, the FAI was significantly higher in patients with reduced CFR than in those with normal CFR among patients without obstructive CAD. The FAI was independently related to reduced CFR among patients without obstructive CAD. Patients with microvascular dysfunction but without epicardial coronary stenosis showed a higher FAI. Moreover, a higher FAI was associated with reduced CFR after adjusting for the risk factors for CAD and the presence of obstructive CAD and DA. The results suggest that FAI is an independent factor for predicting reduced CFR among patients with suspected CAD. In addition, peri-coronary inflammation was associated with decreased stress MBF, which eventually affected CFR. To the best of our knowledge, this is the first study to investigate the relationship between coronary vascular function (particularly microcirculation function) evaluated by CFR and peri-coronary inflammation assessed by CCTA among many patients with suspected CAD.

Currently, most studies have focused on the effect and prediction of peri-coronary inflammation on the hemodynamics of epicardial coronary arteries; however, studies on the effect of coronary vascular functions, particularly microvascular functions, are lacking. Only one study has reported a correlation between CFR and peri-coronary inflammation, suggesting that PCAT attenuation may help identify myocardial ischemia specifically in patients who are traditionally considered not at a high risk of future cardiovascular events (13). However, this previous study included a relatively small sample size and did not include patients with obstructive CAD. Thus, further larger studies should be performed to verify differences in this subset in order to validate the relationship between FAI and microvascular function. Nevertheless, the present study compensated for the aforementioned limitations to some extent. Masahiro et al. (16) found that microcirculation dysfunction was not significantly correlated to the FAI and epicardial fat attenuation; however, it was significantly correlated with epicardial fat volumes in patients with moderate coronary stenosis (30%–80%) when invasive FFR and IMR values were considered as the gold standard. Herein, the reduced CFR group showed a higher FAI in patients without obstructive CAD, which indicated the association between microvascular dysfunction and FAI. However, no significant difference in FAI was observed between the reduced CFR group and the patient group without obstructive CAD. We consider that two possible reasons are the individual variations in FAI and a small sample size. Expanding the sample size would be valuable to enhance the precision of the conclusion.

PCAT supports and protects the surrounding coronary vessels. Previous studies have confirmed that FAI reflects peri-coronary inflammation and is associated with vulnerable plaques and inflammatory activities (1, 17). Moreover, the paracrine-mediated regulation of vascular homeostasis by PCAT has been reported in a study (18). In cases of CAD or microvascular dysfunction, radical oxygen species generated in response to regional ischemia can trigger the activation of EAT-driven inflammatory signals by releasing chemokines and inflammatory cytokines, which exert local effects on the underlying coronaries and myocardium (18). The possible mechanism is that coronary artery inflammation in patients with impaired coronary blood flow may disrupt the role of PCAT in regulating vascular tension caused by focal inflammation. Siasos et al. (19) reported an association between microvascular and epicardial endothelium dysfunction and local lower endothelial shear stress (ESS) during early-stage coronary atherosclerosis. Low ESS, the tangential stress produced by the friction of the circulating blood with the endothelial surface, is a focal proinflammatory stimulus associated with coronary atherosclerosis development and progression (19). Another probable reason for increased FAI in patients with reduced CFR is that microvascular endothelial dysfunction may reversibly decrease upstream local ESS, thereby exacerbating the inflammation of the upstream coronary artery endothelium. A bidirectional pathway between the vascular wall and the surrounding fat and the secretion of cytokines and inflammatory mediators may increase the density of peri-coronal fat (20).

Decreased CFR represents focal vascular stenosis and impaired vasodilation caused by diffused atherosclerosis. Similarly, herein, both obstructive CAD and DA were associated with reduced CFR. DA is related to a worse prognosis, irrespective of focal stenosis severity, and localized revascularization by stent insertion may not completely restore the hemodynamics impaired by DA (7), which suggests that attention should be paid to DA when evaluating the coronary circulation of patients with suspected CAD. Previous studies have shown the effects of CTA plaque characteristics on downstream myocardial perfusion evaluated by PET. A high disease burden was predicted by CFR and adverse plaque by hyperemic flow and resting flow. High-risk plaque characteristics and plaque burden were independently associated with cardiovascular events (21). Driessen et al. (22) showed that positive remodeling and non-calcified plaque volumes were related to detrimental downstream hyperemic blood flow in 208 patients with suspected CAD. They confirmed that compared with patients with normal CFR, patients with reduced CFR showed a significantly higher prevalence of low-attenuation plaques and positive remodeling, which is in line with the results of our study.

Nevertheless, the present study has several limitations. First, we included limited single-center retrospective data. Second, high-risk plaque characteristics were considered in the study, and plaque parameters such as plaque volumes and lesion lengths were not quantitatively measured. Third, the absence of epicardial diseases was presumed based on anatomical obstructive lesions owing to the lack of FFR data, which did not necessarily indicate true physiological microvascular dysfunction. Fourth, the absolute difference in FAI between the normal CFR group and the reduced CFR group was minuscule, which indicated that applying the index to clinical practice would be challenging. Hence, more studies should be performed to collect multicenter prospective data and quantitatively evaluate lesion parameters.

## Conclusion

In conclusion, coronary perivascular inflammation evaluated by CCTA was found to be independently related to coronary vascular function. FAI was higher in patients without obstructive CAD in the presence of reduced CFR. Thus, FAI may help predict microcirculatory damage among patients who do not show epicardial artery stenosis.

## Data Availability

The raw data supporting the conclusions of this article will be made available by the authors, without undue reservation.
